# Supplementary material for: Activation of Anti-SARS-CoV-2 Human CTLs by Extracellular Vesicles Engineered with the N Viral Protein
Source: Vaccines (Basel). 2022 Jun 30;10(7):1060. doi: 10.3390/vaccines10071060 (PMC9318727; doi:10.3390/vaccines10071060)
Supplement: Supplementary file 1 [file vaccines-10-01060-s001.zip › vaccines-1762816-supplementary.pdf]

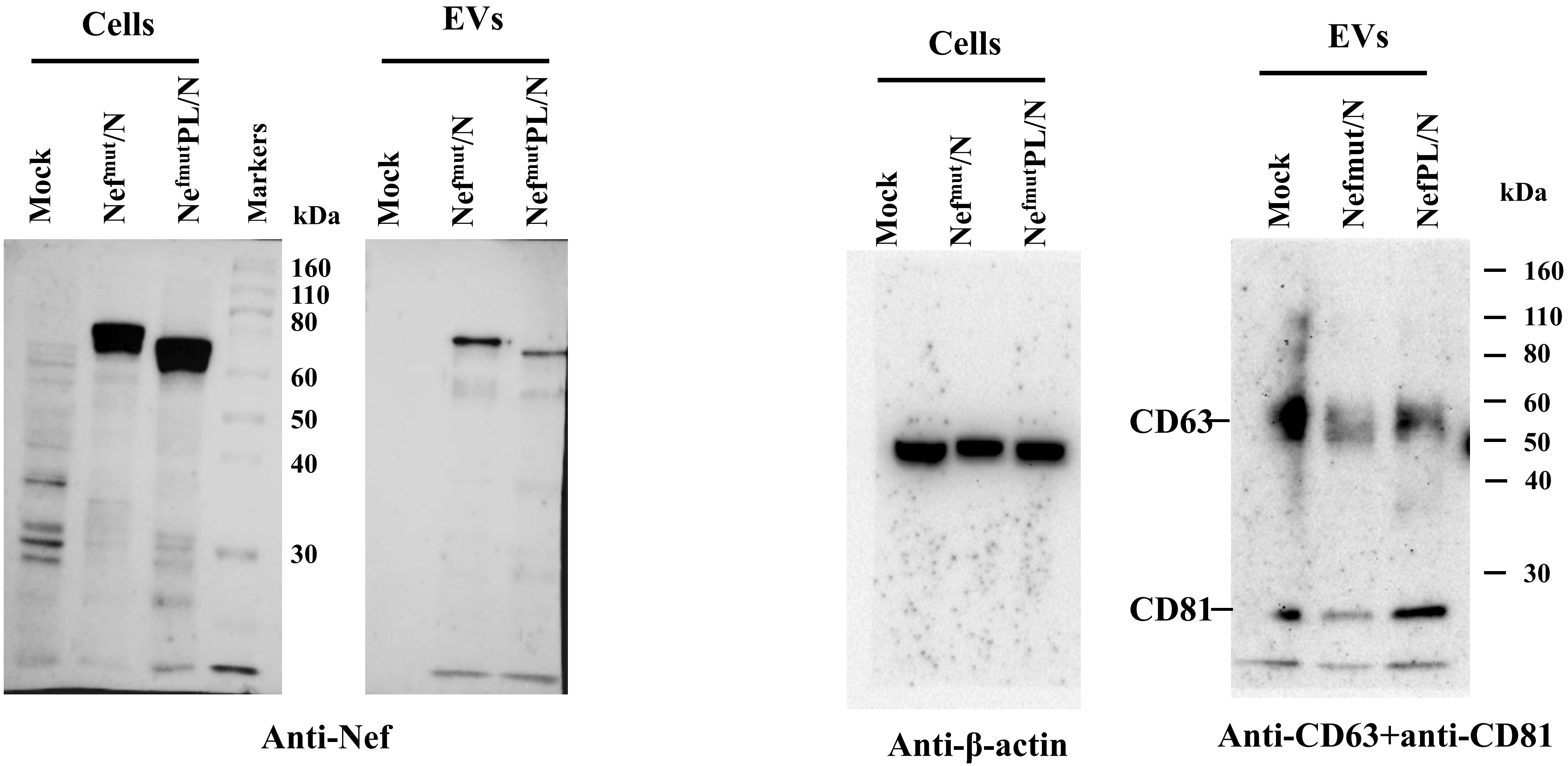

Supplementary Figure S1. Western blot analysis of transfected cells and EVs isolated from respective supernatants: raw data.

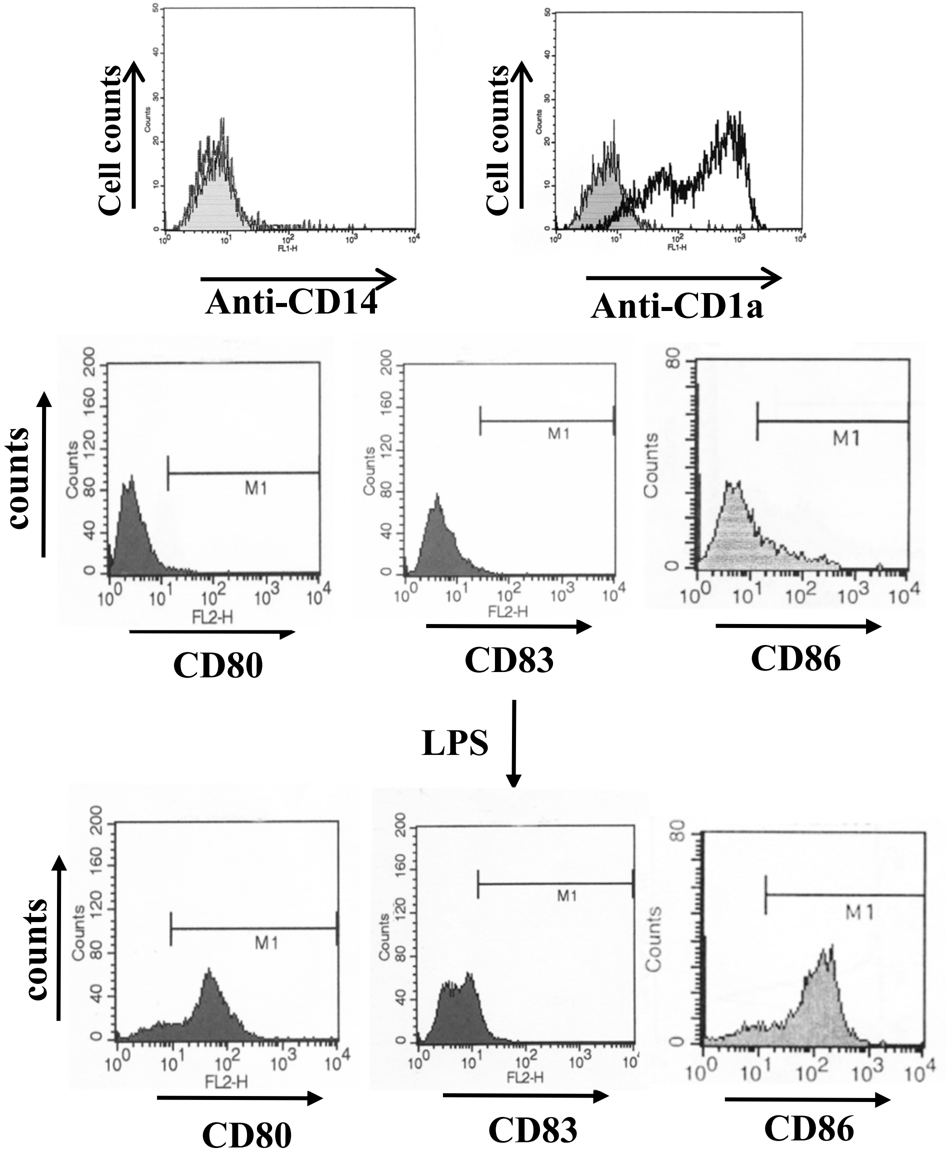

Supplementary Figure S2. Phenotype analysis of PBMC-derived monocytes after 5 days of cultivation in the presence of both GM-CSF and IL-4. Cells were tested for the expression of CD14 (i.e., marker of monocyte-macrophages), the DC-related CD1a, and, also after LPS-treatment, CD80, CD83, and CD86 markers. M1 signs the range of positivity. The data are representative of five independent experiments.

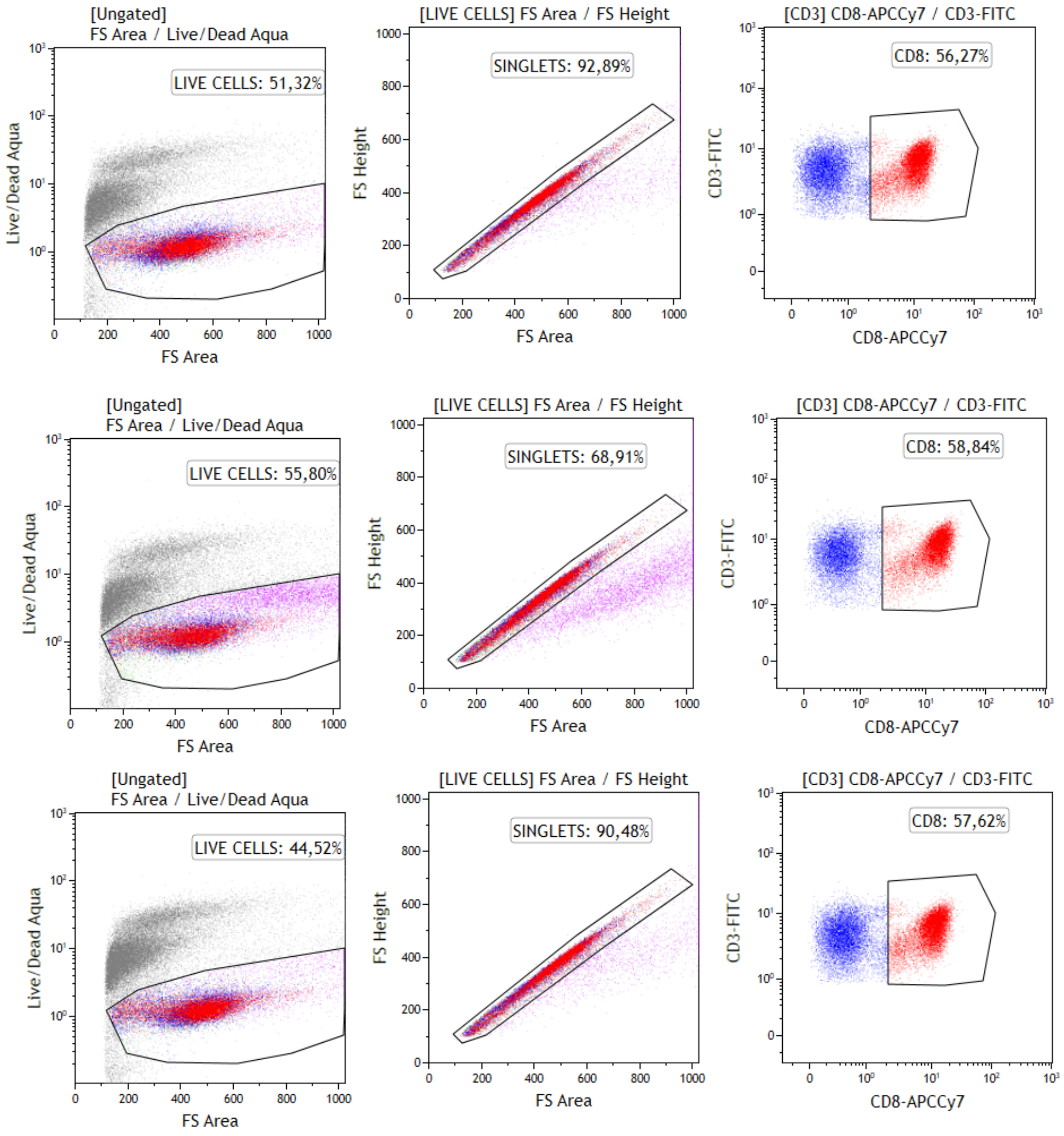

Supplementary Figure S3. Gating strategy applied on PBLs isolated from co-cultures with challenged DCs before analysis of either CD107a or CM-Dil positive cells.
